# Improved Iron Uptake and Metabolism Through Combined Heme and Non-Heme Iron Supplementation: An In Vitro Study

**DOI:** 10.3390/biomedicines14010043

**Published:** 2025-12-24

**Authors:** Francesca Parini, Rebecca Galla, Simone Mulè, Matteo Musu, Francesca Uberti

**Affiliations:** 1 Noivita Srls, Spin Off, University of Piemonte Orientale (UPO), Strada Privata Curti 7, 28100 Novara, Italy; francescaparini00@gmail.com (F.P.); rebecca.galla@noivita.it (R.G.); 2 Laboratory of Physiology, Department for Sustainable Development and Ecological Transition, University of Piemonte Orientale (UPO), Piazza Sant’Eusebio 5, 13100 Vercelli, Italy; simone.mule@uniupo.it (S.M.); 20049586@studenti.uniupo.it (M.M.)

**Keywords:** iron supplementation, non-heme iron, heme iron, dual-source iron, intestinal absorption, 3D in vitro intestinal barrier, iron transporters

## Abstract

Iron is essential for numerous physiological processes, including oxygen transport, energy metabolism, and immune function. This study evaluated the efficacy and safety of three iron formulations combining heme and non-heme iron, comparing them with existing market products and the original form of iron. The formulations tested were GlobiFer^®^ Forte, a combination of heme and non-heme iron containing 18 mg of elemental iron (hereinafter referred to as nutraceutical product 1); GlobiFer^®^, a combination of heme and non-heme iron containing 14 mg of elemental iron (hereinafter referred to as nutraceutical product 2); and a double dose of nutraceutical product 2. Using an in vitro 3D intestinal barrier model, all three formulations significantly increased tight junction protein expression and TEER values, indicating preserved barrier integrity. Iron absorption analysis revealed that all three iron formulations had higher absorption rates than controls. Nutraceutical product 1 showed the highest absorption, associated with increased expression of the iron transporters such as the primary non-heme iron transporter, DMT1, and the leading apical heme transporter, HCP-1. All three new formulations increased ferritin and ferroportin levels, markers of systemic iron storage and regulation. Nutraceutical product 1 was found to be the most effective, based on percentage. Overall, combining heme and non-heme iron improved intestinal absorption and supported iron metabolism, with Nutraceutical Product 1 proving the most promising in terms of efficacy and safety. These results support the development of optimised dual-source iron supplements to improve bioavailability and maintain intestinal barrier integrity, prerequisites for better efficacy and tolerability in clinical use.

## 1. Introduction

Iron deficiency and anaemia, the most common nutritional deficiency globally, affects approximately 30% of the population, particularly children, women, and adult men, depending on socioeconomic status and health conditions [1]. A lack of both quantitative and qualitative iron in the diet is frequently the cause of anaemia, a condition in which the body’s red blood cells are insufficient to meet physiological demands [2]. Iron is crucial for human growth, particularly in children, as it significantly impacts their development. Furthermore, it is essential for brain growth, myelination, monoamine neurotransmitter action, and the energy metabolism of neurons and glial cells [3]. Shortness of breath, exhaustion, palpitations, tachycardia, and angina are all signs of iron deficiency, which is caused by low levels of iron in the blood. Abdominal pain, nausea, weight loss, intestinal blood flow problems, and motility disorders can all result from this hypoxemia [1].

The World Health Organisation (WHO) defines anaemia as a haemoglobin concentration of less than 130 g/L for men, less than 120 g/L for non-pregnant women, and less than 110 g/L for pregnant women, regardless of trimester [4]. On the other hand, iron deficiency is characterised by plasma ferritin levels below 12 μg/L (not adjusted for infection or inflammation), serum ferritin levels below 15 μg/L, and transferrin saturation below 10% [5]. The terms iron deficiency and anaemia are often used interchangeably today; however, these terms are distinct, as iron deficiency precedes anaemia, and if left untreated, anaemia will develop [6]. Conversely, high iron exposure can overwhelm compensatory mechanisms, leading to iron accumulation and toxicity. As a result, oxidative stress and damage are produced, mostly in mitochondria. Excessive iron deposition can lead to severe diseases, including impaired β-cell function (diabetes) and increased iron storage in mitochondria, resulting in oxidative damage to cardiomyocytes (contributing to heart failure) [7].

Iron levels in the body are primarily controlled by dietary intake, intestinal absorption, and iron recycling [8]. Iron from diet can be subdivided into two forms: heme and non-heme iron. Heme iron is highly absorbable and is found in animal products such as meat, poultry, and fish. Non-heme iron is mostly found in plant food but is less absorbable due to compounds like phytate, oxalate, polyphenols, and tannin. Conversely, ascorbic acid, citrate, and gastric acid facilitate iron absorption [9]. It is important to consider that a healthy diet should provide 5–15 mg of elemental iron and 1–5 mg of heme iron daily, of which only 1–2 mg is absorbed into the intestine [10]. In humans, an active, carrier-mediated mechanism involving a membrane iron-binding glycoprotein facilitates iron uptake in the upper small intestine. Because iron lacks active excretory systems, intestinal absorption plays a major role in maintaining body iron homeostasis in humans [11]. Ferrous iron is taken up at the brush border of duodenal enterocytes via divalent metal transporter 1 (DMT1) [12]. In contrast, heme dietary iron is transported into the enterocytes by heme carrier protein-1 (HCP-1); here, heme iron is converted into ferrous iron in a reaction catalysed by heme oxygenase [13]. Once inside the enterocyte, iron is exported across the basolateral membrane into the portal circulation by ferroportin. In the bloodstream, iron binds to transferrin, which delivers it to target cells, such as those in the bone marrow, liver, and macrophages, via transferrin receptor 1-mediated uptake [12]. Sweating, menstruation, and the shedding of skin and hair cells all contribute to uncontrolled iron excretion, which involves enterocyte excretion and rapid turnover [14]. For intestinal absorption, dietary non-heme iron (Fe^3+^) must first be converted to Fe^2+^ (ferrous iron). DMT1 enables this reduced iron to enter the body through the apical membrane of the enterocytes. Enterocytes can either release iron across the basolateral membrane for systemic distribution, store it in ferritin, or use it directly for intracellular metabolic processes [15]. Following absorption in enterocytes, ferroportin transports reduced iron (Fe^2+)^ to the circulation; once oxidised by ceruloplasmin or hephaestin to Fe^3+^, the iron attaches to transferrin, the primary plasma iron carrier, for subsequent use [16]. The hepcidin/ferroportin axis regulates iron homeostasis at the systemic level [17]. Iron deficiency, hypoxia, and erythropoietic expansion (also known as stress erythropoiesis) all result in decreased hepcidin expression [18]. Furthermore, iron deficiency reduces ferroportin translation, helping cells conserve iron by limiting its export. At the same time, transferrin levels rise to maximise iron transport efficiency in the circulation. This coordinated response enhances the body’s ability to capture, retain, and redistribute the limited available iron, ensuring that essential processes such as erythropoiesis can continue despite reduced iron stores [6,7].

Improved consumption of some micronutrients, such as iron, folate, and vitamin B12, through dietary variety, food fortification, and supplementation is one of the primary objectives of the WHO’s current policy to combat iron deficiency [19]. Iron salts have traditionally been prescribed as the first-line treatment for iron-deficient anaemia at doses of 100–200 mg per day. However, current knowledge suggests that the actual absorbed dose is significantly lower because the same iron entering the bloodstream raises serum iron levels, which in turn raise the production of hepcidin, the master regulator of iron absorption and transport, which restricts the amount of iron that is absorbed from the gut. Unabsorbed iron in the intestinal lumen generates reactive oxygen species, which cause tissue damage and are responsible for the most prevalent side effects, such as nausea, vomiting, constipation or diarrhoea, abdominal discomfort, and black stool [6].

Heme iron supplementation represents a particularly promising strategy, as it provides substantially higher bioavailability than conventional iron salts. Unlike non-heme iron, heme iron is absorbed as an intact metalloporphyrin through a distinct and more efficient intestinal pathway likely mediated by HCP1 and endocytic processes, which makes it far less susceptible to dietary inhibitors and variations in gastrointestinal conditions. After internalisation, heme is enzymatically degraded by heme oxygenase within enterocytes, releasing ferrous iron that is readily mobilised into the systemic circulation. Owing to this highly efficient mechanism, clinical intervention studies consistently show that heme iron achieves superior haemoglobin regeneration and more robust improvements in overall iron status compared with inorganic iron formulations [20,21]. Even if heme and non-heme iron are absorbed in the intestines by two distinct molecular processes, the iron is stored in the iron storage protein. It enters the same intracellular pool as freshly absorbed heme or non-heme iron [10]. However, heme iron may have a bigger effect on iron status markers, according to bioavailability research and observational data [20].

Based on the current literature, we hypothesised that formulations containing different dosages of heme and non-heme iron would lead to better iron absorption and metabolism than formulations containing only non-heme iron. The aim was to evaluate whether the combined use of these two iron forms could enhance ferritin levels and to determine which formulation is more effective at compensating for iron deficiency. Further, the study aimed to evaluate, in vitro, the different absorption rates and the impact of the formulations on iron metabolism. The data obtained were compared with those of commercial products to evaluate the possible improved efficacy of the combination with heme iron.

## 2. Materials and Methods

### 2.1. Agent Preparation

GlobiFer^®^ forte, a combination of heme and non-heme iron containing 18 mg of elemental iron (hereinafter referred to as nutraceutical product 1), GlobiFer^®^, a combination of heme and non-heme iron containing 14 mg of elemental iron (hereinafter referred to as nutraceutical product 2), and a double dose of nutraceutical product 2 (donated from Ceres Pharma, Ghent, Belgium) were prepared in Dulbecco’s Modified Eagle’s Medium (DMEM, Merck Life Science, Rome, Italy). It contained no phenol red and was supplemented with 1% penicillin-streptomycin, 0.5% foetal bovine serum (FBS), and 2 mM L-glutamine (all from Merck Life Science, Rome, Italy). All analyses were performed and compared to different commercial products, specifically one product containing 14 mg of ferric iron in the form of sucrosomal iron (referred to as commercial product 1), 30 mg of ferric iron in the form of sucrosomal iron (referred to as commercial product 2), and 80 mg ferrous iron as iron sulphate (referred to as commercial product 3). Further, the analysis was performed in comparison with two raw materials, namely iron sulphate and iron bisglycinate. The agents contained different iron concentrations, so they were normalised to 1 mg/mL.

### 2.2. Cell Culture

The Caco-2 cell line, derived from human Caucasian colon adenocarcinoma, was acquired from the American Type Culture Collection (ATCC, Manassas, VA, USA). This cell line was cultivated in DMEM Advance (DMEM-Adv, Thermo Fisher Scientific, Rodano, MI, Italy) with the addition of 10% FBS (Merck Life Science, Rome, Italy), 2 mM L-glutamine, and 1% penicillin-streptomycin (all from Merck Life Science, Rome, Italy). The cells were maintained in an incubator at 37 °C and with 5% CO_2_. The use of this cell line in experimental models that predict drug absorption, metabolism, and bioavailability following oral delivery has been licenced by the Food and Drug Administration (FDA) and the European Medicines Agency (EMA) [22,23,24]. For the experiments, 2 × 10^4^ cells were plated in a 24-well plate using 6.5 mm Transwell^®^ inserts and a polycarbonate membrane with 0.4 μm pores (CorningCostar, New York, NY, USA). Before stimulation, the cells were maintained for eight hours in an incubator using DMEM-Adv medium devoid of red phenol and supplemented with 0.5% FBS, 2 mM L-glutamine, and 1% penicillin-streptomycin (all from Merck Life Science, Rome, Italy) [22].

### 2.3. Experimental Protocol

To evaluate the effects of different forms of iron on 3D in vitro intestinal barrier, different analyses were performed. Specifically, three different formulations of iron sulphate and heme iron were analysed. Firstly, in vitro pre-digestion was performed using a mixture of sample and pepsin at a mass ratio of 1:20 *w*/*w* in a pH 2 stimulation medium (to which HCl has been added; Merck Life Science, Rome, Italy) to mimic gastric digestion. Then, the integrity of the intestinal barrier was assessed using trans-epithelial electrical resistance (TEER) and tight junction analyses. Then, the kinetics of absorption were analysed to understand the quantity of iron that crosses the intestinal barrier. Finally, the effects of different iron samples on specific markers of iron metabolism were analysed, namely DMT-1, ferroportin and ferritin. Furthermore, to clarify whether heme iron plays a greater role in the mechanisms related to iron absorption, HCP-1, a heme-iron receptor, was also analysed.

### 2.4. In Vitro Intestinal Barrier Model

A standard approach for assessing the in vitro absorption, metabolism, and bioavailability of a compound administered orally has been approved by the FDA and EMA. Transwell^®^ systems were used to recreate this in vitro model of the intestinal barrier. Precisely, 2 × 10^4^ Caco-2 cells were seeded onto Transwell^®^ membrane and maintained in complete medium for 21 days before stimulation [22]. At around 21 days, the TEER of Caco-2 cells was measured using EVOM3™ with STX2 chopstick electrodes (World Precision Instruments, Sarasota, FL, USA) to assess the maturity of the intestinal epithelial formation and the effectiveness of paracellular mechanisms; the indicative TEER value had to be 400 Ω∙cm^2^ [25]. Before stimulation, the medium on the apical side was adjusted to a pH of 6.5 to replicate the intestinal lumen environment; on the basolateral side, blood conditions were simulated at a pH of 7.4 [26]. A Transwell^®^ with the same growth medium as the cells, but without cells, was used to eliminate background noise from the semi-porous membrane, allowing for accurate determination of TEER values.

After every stimulation, TEER measurements were taken to monitor any potential degradation on the apical side. A fluorescent tracer at 0.04% (Santa Cruz, CA, USA) was used to measure compound uptake at each time point [27]. Caco-2 cells were incubated for 40 min at the specified concentration to determine the amount of fluorescein transported at 37 °C. A fluorescence spectrophotometer (Infinite 200 Pro MPlex, Tecan, Männedorf, Switzerland) was used to measure fluorescence at excitation/emission wavelengths of 490/514 nm, respectively. The permeation rate in % is determined using the equation below [22]:

Papp = dQ/dt ⇥ 1/m0 ⇥ 1/A ⇥ V Donor

dQ: amount of substance transported (nmol or μg);
dt: incubation time (s);
m0: amount of substrate applied to the donor compartment (nmol or μg);
A: Transwell^®^ membrane surface area (cm^2^);
V Donor: volume of the donor compartment (cm^3^).

The results were expressed as mean ± SD (%), and negative controls without cells were tested to exclude the influence of the Transwell^®^ membrane.

### 2.5. Tight Junction (TJ) Analysis

The levels of Occludin, Claudin-1 and Zonula Occludens-1 (ZO-1) were analysed in CaCo-2 lysates using, respectively, Human Occludin (OCLN) ELISA Kit (MyBiosource, San Diego, CA, USA), Human Claudin-1 (CLDN1) ELISA kit (Cusabio Technology LCC, Huston, Houston, TX, USA) and the human tight junction protein 1 (TJP1) ELISA kit (MyBiosource, San Diego, CA, USA) following the manufacturer’s instruction [28]. The absorbance was measured by using a spectrophotometer (Infinite 200 Pro MPlex, Tecan, Männedorf, Switzerland) at 450 nm. The data were obtained by comparing them to the standard curve (from 0 to 1500 pg/mL for occludin, from 0 to 1000 pg/mL for claudin-1 and ZO-1). The results were then expressed as a percentage (%) vs. the control (0 line) of five separate experiments in triplicate.

### 2.6. Iron Quantification Assay

According to the manufacturer’s instructions, an Assay Kit (Merck Life Science, Rome, Italy) was used to quantify iron [29]. The basolateral medium (supernatant) environment was used for the measurements. At each time point, a spectrometer (Infinite 200 Pro MPlex, Tecan, Männedorf, Switzerland) was used to measure absorbance at 593 nm. The results of five tests, each carried out in triplicate, were then presented as averages (%) relative to the control (0 lines).

### 2.7. DMT1 ELISA Kit

DMT1 presence was assessed using the Human Divalent Metal Transporter 1 (DMT1) ELISA Kit (MyBioSource, San Diego, CA, USA) according to the manufacturer’s instructions [30]. The absorbance was measured using a spectrophotometer (Infinite 200 Pro MPlex, Tecan, Männedorf, Switzerland) set at 450 nm. The concentration was expressed as ng/mL, with data compared to a standard curve (0 to 50 ng/mL). The results were presented as means (%) versus the control (0 line) of five independent experiments conducted in triplicate.

### 2.8. Western Blot

Transwell systems were lysed in ice with Complete Tablet Buffer (Roche, Basilea, Switzerland) supplemented with 2 mM sodium orthovanadate (Na_3_VO_4_), 1 mM phenylmethanesulfonylfluoride fluoride (PMSF, Merck Life Science, Rome, Italy), a 1:50 mix of Phosphatase Inhibitor Cocktail (Merck Life Science, Rome, Italy), and a 1:200 mix of Protease Inhibitor Cocktail (Merck Life Science, Rome, Italy). Additionally, the cells on the Transwell were scraped, and the cellular components were homogenised with lysis buffer using a scraper. The entire mixture was then resuspended using a tip and moved to a chilled Eppendorf tube. The pellets and supernatants were separated using an Eppendorf tube centrifuge set to 13.500 rpm and 4 °C for 30 min. A bicinchoninic acid assay was used to measure the amount of total protein in the sample after the supernatant was transferred into a fresh Eppendorf (BCA, Thermo Fisher, Waltham, MA, USA). After taking 2 μL of each sample and dispensing it into a 96-well multiwell, 200 μL of BCA solution was added. A spectrophotometer (Infinite 200 Pro MPlex, Tecan, Männedorf, Switzerland) was used to measure the plate’s absorbance at 562 nm after incubation for 30 min at 37 °C in the dark. Concurrently, a calibration curve was created to determine the protein content of the test samples using Bovine Serum Albumin (BSA) 2 mg/mL at progressively higher concentrations. The protein content of each sample was calculated by interpolating the calibration line and expressed as μg/μL. According to the standard protocol, 30 μg of protein from each sample was resolved on 10% SDS-PAGE gels, and polyvinylidene difluoride (PVDF) membranes (GE Healthcare Europe GmbH, Milan, Italy) were incubated overnight at 4 °C with the primary antibody anti-HCP-1 (1:500; Santa Cruz, CA, USA). Protein expression was normalised and verified through anti-β-actin detection (1:5000, Merck Life Science, Rome, Italy). The results were expressed as means ± SD (% vs. control).

### 2.9. Ferritin ELISA Kit

The Human Ferritin ELISA kit (Abcam, Cambridge, UK) was used to measure ferritin levels in cell lysates, following the manufacturer’s protocols [31]. A spectrophotometer (Infinite 200 Pro MPlex, Tecan, Männedorf, Switzerland) was used to measure the absorbance at 450 nm. Lastly, a standard curve ranging from 0 to 50 ng/mL was used to determine the concentration. The results of five tests, each carried out in triplicate, were then presented as averages (%) relative to the control (0 lines).

### 2.10. Ferroportin ELISA Kit

A specific ELISA kit (Cloud-Clon Corp., Houston, TX, USA) was used to measure the amount of ferroportin in cell lysates in accordance with the manufacturer’s protocols [32], and a spectrophotometer (Infinite 200 Pro MPlex, Tecan, Männedorf, Switzerland) was used to measure the absorbance at 450 nm. Lastly, a standard curve ranging from 0 to 20,000 ng/mL was used to convert concentrations to pg/mL. The results were then presented as averages (%) across five tests, each carried out in triplicate, relative to the control (0 lines).

### 2.11. Statistical Analysis

The one-way analysis of variance (ANOVA) and Bonferroni post hoc tests were used to analyse the data in the statistical programme Prism GraphPad version 9.4.1 (GraphPad Software, Inc., San Diego, CA, USA). A two-tailed Student’s *t*-test was used to compare the two groups. A two-way ANOVA and a two-sided Dunnett post hoc test were used for multiple group comparisons. Every result was presented as the mean ± SD, based on at least 5 independent experiments performed in triplicate.

## 3. Results

### Examination of Intestinal Effects Using Intestinal Barrier Model to Investigate Integrity and Absorption

The first experiments used a 3D in vitro intestinal barrier model to evaluate the ability of different iron formulations to maintain epithelial integrity.

By measuring TEER values over a 1 to 6 h treatment period and evaluating TJ protein levels at the end of the 6 h treatment, the experiments conducted on this model enabled assessment of the intestinal barrier’s integrity. TJ proteins, including occludin, ZO-1, and claudin-1, are members of the multiprotein junctional complex that regulates the movement of ions, water, and solutes via the paracellular pathway, which, in turn, controls the function of the intracellular barrier [33].

**Figure 1:**
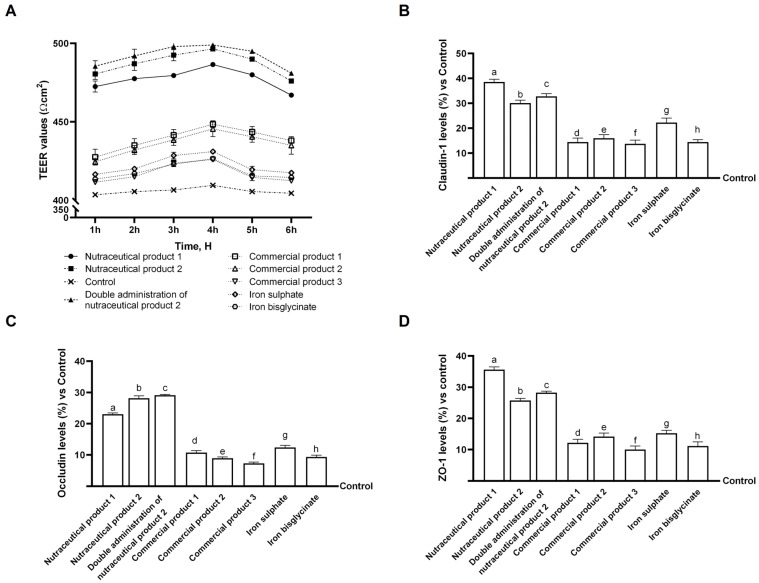
Integrity analysis of the intestinal barrier in vitro. (**A**) TEER value assessed using EVOM3™; (**B**–**D**) study of tight junctions via ELISA tests for claudin-1, occludin and ZO-1. Five separate tests were conducted in triplicate, and the results are presented as means ± SD (%), compared to control values (represented by the 0% line). In (**A**), nutraceutical product 1 *p* < 0.0001 vs. control; nutraceutical product 2 *p* < 0.0001 vs. control; Double administration of nutraceutical product 2 *p* < 0.0001 vs. control; Commercial product 1 *p* = 0.0004 vs. control; Commercial product 2 *p* = 0.0006 vs. control; Commercial product 3 *p* = 0.0162 vs. control; Iron sulphate *p* = 0.0029 vs. control; Iron bisglycinate *p* = 0.0034 vs. control. In (**B**) all *p* < 0.0001 vs. control; a *p* < 0.0001 vs. b,c,d,e,f,g,h; b *p* < 0.0001 vs. d,e,f,g,h; c *p* < 0.0001 vs. d,e,f,g,h; d *p* = 0.004 vs. g; e *p* = 0.0042 vs. g; f *p* = 0.0004 vs. g. In (**C**) all *p* < 0.0001 vs. control; a *p* < 0.0001 vs. b,c,d,e,f,g,h; b *p* < 0.013 vs. d,e,f,g,h; c *p* < 0.0018 vs. d,e,f,g,h; d *p* = 0.006 vs. g; e *p* = 0.0233; f *p* = 0.0035 vs. g; g *p* = 0.0061 vs. h. In (**D**) all *p* < 0.0001 vs. control; a *p* < 0.001 vs. b,c,d,e,f,g,h; b *p* < 0.0001 vs. d,e,f,g,h; c *p* < 0.0001 vs. d,e,f,g,h; e *p* = 0.0375 vs. f; f *p* = 0.0103 vs. g; g *p* = 0.0428 vs. h.

As illustrated in Figure 1A, all agents exert a positive effect on cell integrity compared to the control throughout the treatment period (*p* < 0.05). The results obtained demonstrated that nutraceutical product 1, nutraceutical product 2 and the double administration of nutraceutical product 2 exerted the most significant effects compared to the other agents examined (*p* < 0.05). Furthermore, although the trends among these three formulations were quite similar, with a peak at 4 h, the nutraceutical product 2 and its double administration exerted better effects regarding TEER values (*p* < 0.05). As illustrated in Figure 1B–D, the analysis of three different TJs also demonstrated that all tested substances could preserve epithelial integrity compared to the control (*p* < 0.05). Specifically, ZO-1, which simultaneously maintains and modifies barrier integrity; occludin, which aids in stabilising and optimising barrier function; and claudin-1, the primary barrier-forming protein, were examined. In all the analyses, the best effects were obtained following stimulation with the 3 formulas containing heme iron (*p* < 0.05). The results shown in Figure 1C demonstrated that nutraceutical product 2 and its double administration induced the most beneficial effects regarding occludin levels compared to all the other agents under examination (respectively, about 22% and 26.5% vs. nutraceutical product 1, 1.6-fold and 1.7-fold vs. commercial product 1, 2.2-fold and 2.3-fold vs. commercial product 2, 2.9-fold and 3-fold vs. commercial product 3, 1.3-fold and 2-fold vs. iron sulphate, 1.4-fold and 2.1-fold compared to iron bisglycinate, *p* < 0.05). Different data were obtained for Claudin-1 analysis as the most positive effects were exerted by nutraceutical product 1 compared to all the other agents (about 28% vs. nutraceutical product 2, 17% vs. double administration of nutraceutical product 2, 1.6-fold vs. commercial product 1, 1.4-fold vs. commercial product 2, 1.8-fold vs. commercial product 3, 73% vs. iron sulphate, 1.7-fold vs. iron bisglycinate, *p* < 0.05). Similarly, also in Zo-1 analyses nutraceutical product 1 induced the strongest effects compared to all the other agents (about 38% vs. nutraceutical product 2, 26% vs. double administration of nutraceutical product 2, 1.9-fold vs. commercial product 1, 1.5-fold vs. commercial product 2, 2.6-fold vs. commercial product 3, 1.3-fold vs. iron sulphate and 2.2-fold vs. iron bisglycinate, *p* < 0.05).

**Figure 2:**
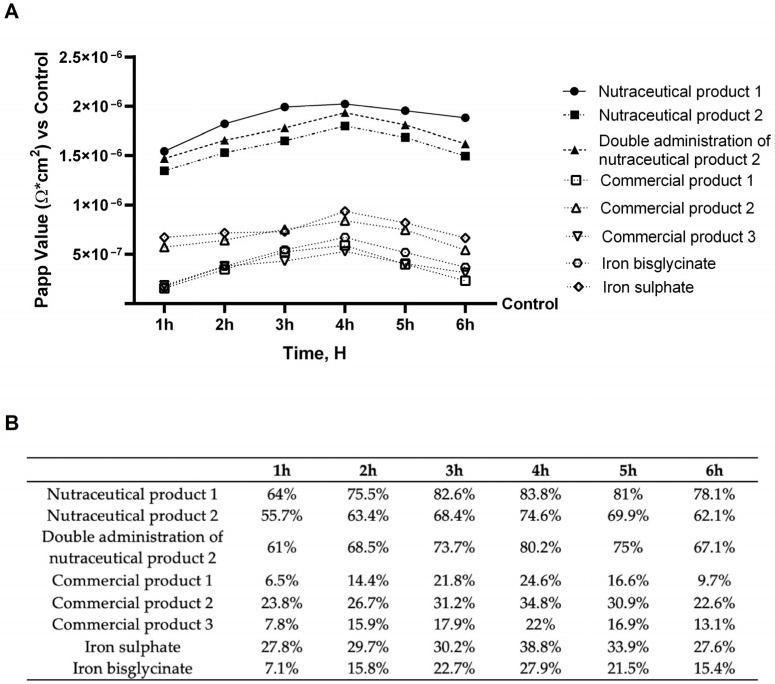
Absorption analysis on the intestinal barrier in vitro. (**A**) Evaluation of passage through the intestinal barrier using a fluorescent tracer. (**B**) Total iron quantification with a specific ELISA kit. Five separate tests were conducted in triplicate, and the results are presented as means ± SD (%), with control values shown in A (the 0% line). Nutraceutical product 1 *p* < 0.0001 vs. control; nutraceutical product 2 *p* < 0.0001 vs. control; Double administration of nutraceutical product 2 *p* < 0.0001 vs. control; Commercial product 1 *p* = 0.0004 vs. control; Commercial product 2 *p* < 0.0001 vs. control; Commercial product 3 *p* = 0.0004 vs. control; Iron sulphate *p* < 0.0001 vs. control; Iron bisglycinate *p* < 0.0001 vs. control.

Experiments were conducted to evaluate the agents that yielded the best absorption results. In Figure 2A, the absorption spectra of the different agents are shown. The data obtained demonstrate that the agents have a higher absorption rate than the control; among them, nutraceutical product 1, nutraceutical product 2, and the double administration of nutraceutical product 2 have the best absorption profile compared to the other agents (*p* < 0.05). Specifically, nutraceutical product 1 demonstrated a higher absorption profile than the other two formulas tested (*p* < 0.05). Nutraceutical product 2 and its double administration demonstrated a similar profile, with a slightly higher absorption rate in the double administration. More precise data were obtained using a specific total iron detection kit to more correctly assess the amount of iron that has crossed the intestinal barrier. As shown in Figure 2B, nutraceutical product 1 induced the strongest effects, with a plateau of approximately 83% between 3 h and 4 h. For all other agents, the peak was observed at 4 h, with similar profiles between commercial product 1 and commercial product 3, and a slightly greater effect with commercial product 2. More precisely, nutraceutical product 1 at 4 h demonstrated an increase in iron absorption of 12% compared to nutraceutical product 2, of 5% compared to double administration of nutraceutical product 2, of 2.4-fold compared to commercial product 1, of 1.4-fold compared to commercial product 2, of 2.8-fold compared to commercial product 3, of 1.2-fold compared to iron sulphate and of 2-fold compared to iron bisglycinate. Further, in contrast to the other substances analysed, which display maximal absorption at 4 h followed by a subsequent decline in absorption rate, nutraceutical product 1 maintains a nearly constant absorption profile beyond 4 h, with only a marginal reduction observed over time.

**Figure 3:**
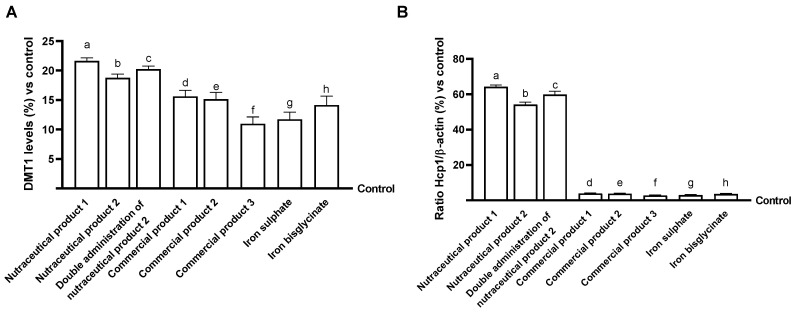

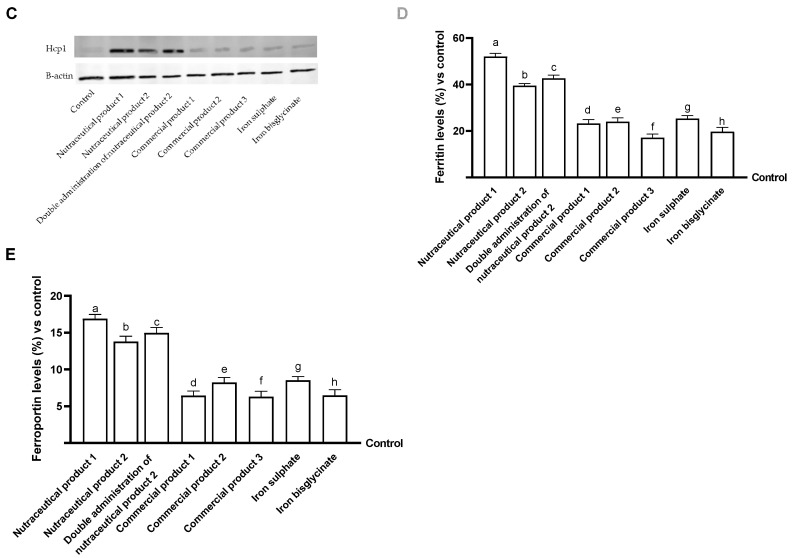
Iron mechanisms on the intestinal barrier in vitro. (**A**) DMT1 analysis via ELISA tests. In (**B**), HCP-1 analysis via Western Blot analysis with an example image of a lane reported in (**C**). In (**D**), ferritin analysis via ELISA tests. In (**E**), and ferroportin analysis via ELISA tests. Five separate tests were conducted in triplicate, and the results are presented as means ± SD (%), compared to control values (represented by the 0% line). In (**A**) all *p* < 0.05 vs. control; a *p* < 0.05 vs. d,e,f,g,h; b *p* < 0.025 vs. e,f,g,h; c *p* < 0.025 vs. d,e,f,g,h; d *p* = 0.0230 vs. f; e *p* = 0.0415 vs. f. In (**B**) a,b,c, *p* < 0.0001 vs. control; a *p* < 0.015 vs. b,c,d,e,f,g,h; b *p* < 0.0032 vs. d,e,f,g,h; c *p* < 0.0001 vs. d,e,f,g,h. In (**D**) all *p* < 0.0001 vs. control; a *p* < 0.02 vs. b,c,d,e,f,g,h; b *p* < 0.0007 vs. d,e,f,g,h; c *p* < 0.0002 vs. d,e,f,g,h; e *p* = 0.0302 vs. f; f *p* = 0.0113 vs. g. In (**E**), all *p* < 0.0001 vs. control; a *p* < 0.023 vs. b,d,e,f,g,h; b *p* < 0.001 vs. d,e,f,g,h; c *p* < 0.0005 vs. d,e,f,g,h.

Finally, the mechanisms concerning iron metabolism were analysed. The effects on DMT1 levels were analysed after stimulation of Caco-2 cells with all agents, as DMT1 is an essential protein for intestinal iron absorption and intracellular transport. As shown in Figure 3A, DMT1 levels increased compared to the control after all stimulations with the agents under examination (*p* < 0.05). In particular, the effects on DMT-1 levels were greater after stimulation with nutraceutical product 1, nutraceutical product 2 and double administration of nutraceutical product 2 (*p* < 0.05 vs. the other agents). Specifically, nutraceutical product 1 induced the highest effects (about 38% vs. commercial product 1, 43% vs. commercial product 2, 97% vs. commercial product 3, 25% vs. iron sulphate and 53% vs. iron bisglycinate, *p* < 0.05), even if not statistically significant compared to nutraceutical product 2 and the double administration. However, the data obtained from analysing this transport protein did not reflect the correct transport of the three heme iron-containing formulations; therefore, subsequent analyses focused on the expression levels of HCP-1, a key protein involved in heme transport, assessed by densitometric quantification of Western blot bands. As shown in Figure 3B, HCP-1 expression was significantly upregulated following stimulation with nutraceutical product 1, nutraceutical product 2, and the double administration of nutraceutical product 2. For completeness, Figure 3C shows a representative Western blot lane illustrating the HCP-1 transporter expression, which served as the basis for the densitometric analysis presented in Figure 3B, thereby supporting the quantitative assessment of its expression levels. These data demonstrate that the iron in these formulations is transported via two distinct mechanisms: one via DMT1 and the other via HCP-1. Finally, the intracellular protein ferritin and the transmembrane protein ferroportin were assessed to better understand the biochemistry of the tested products’ effects on iron metabolism. Iron ions can be extruded from a cell by ferroportin and can be sequestered in a non-toxic form within cells by ferritin. The data obtained on ferritin levels (Figure 3D) confirmed that nutraceutical product 1, nutraceutical product 2, and the double administration treatment induced the most significant effects, compared not only to the control but also to the other agents under investigation. In particular, the effects on ferritin levels were most significant with nutraceutical product 1 (approximately 35% vs. nutraceutical product 2, 26% vs. double administration of nutraceutical product 2, 1.3-fold vs. commercial product 1, 1.2-fold vs. commercial product 2, 2.2-fold vs. commercial product 3, 1.3-fold compared to iron sulphate and 1.7-fold compared to iron bisglycinate, *p* < 0.05). Finally, the analysis of ferroportin levels (Figure 3E), showed that levels increased throughout stimulation with all test agents (*p* < 0.05 compared to control), indicating the operation of an active extrusion mechanism. Once again, the heme iron-containing formulations were superior to the control and the other agents tested (*p* < 0.05). In particular, nutraceutical product 1 stimulated ferroportin levels more effectively than the other products tested (approximately 22% vs. nutraceutical product 2, 11% vs. double administration of nutraceutical product 2, 1.6-fold vs. commercial product 1, 1.1-fold vs. commercial product 2, 1.7-fold vs. commercial product 3, 99% vs. iron sulphate, 1.6-fold compared to iron bisglycinate, *p* < 0.05). Overall, these experiments confirmed that nutraceutical product 1, nutraceutical product 2 and double administration of nutraceutical product 2 are capable of exerting positive effects on iron metabolism, and, in particular, on the main proteins involved in the transport and storage of iron ions in cells.

## 4. Discussion

Iron is vital for mitochondrial and cellular respiration, for the movement and storage of oxygen, and for both cell development and immunological function [34]. Therefore, several detrimental health issues are associated with low iron levels. Usually taken as tablets, capsules or liquids, oral iron therapy is commonly provided in ferrous or ferric forms. Since only about 10% of intestinal iron is normally absorbed, it may take considerable time for the gut to replenish iron levels [35]. Heme iron, a substance present in the protoporphyrin IX ring of haemoglobin and myoglobin, is one potential alternative as it has demonstrated better absorption than non-heme iron [36]. In this regard, this study examined the efficacy of three formulations containing a mixture of heme and non-heme iron compared with existing market products. Specifically, the three formulations analysed were nutraceutical product 1 (combination of heme and non-heme iron containing 18 mg of elemental iron), nutraceutical product 2 (combination of heme and non-heme iron containing 14 mg of elemental iron) and double administration of nutraceutical product 2 (double administration of the combination of heme and non-heme iron containing 14 mg of elemental iron).

The primary location for iron absorption is the gut, which is also susceptible to iron overload. For defence and nutritional absorption, intestinal stability and integrity are essential [37]. For this reason, the first experiments of this study evaluated the integrity of the intestinal barrier, as reproduced in vitro using a 3D model. Even if all the agents under examination were able to increase the TEER values and the levels of claudin, occludin and ZO-1, these data were particularly remarkable when the three formulations containing heme iron were employed. Indeed, nutraceutical product 1, nutraceutical product 2, and the double administration of nutraceutical product 2 induced a strong increase in all these parameters compared to the other agents tested. These data demonstrate that the combination of heme and non-heme iron preserved intestinal integrity and function with greater efficacy, thus highlighting their safety at the intestinal level.

Since humans lack active excretory systems for iron, intestinal absorption is the primary mechanism regulating iron homeostasis. Therefore, lowering the risk of iron deficiency requires effective intestinal iron absorption and utilisation [10]. Experiments were conducted to evaluate iron absorption levels following stimulation with all agents tested. The study focused on the effects of various iron formulations to determine which is most effective in treating iron deficiencies, using a method able to determine both heme and non-heme iron. The results highlighted that nutraceutical treatments significantly increase the availability of heme iron compared to non-heme iron, confirming the absorption and the selective modulation of iron forms. At the same time, based on the findings, it is evident that combining heme and non-heme iron is beneficial because it significantly increases absorption levels, on average, about twice as much as products without heme iron. As a result, using a combination of heme and non-heme iron could resolve the clinic’s absorption issues. Among the three formulations containing both iron types, nutraceutical product 1 showed the highest absorption. The obtained results are correlated with heme iron having higher bioavailability on its own and with its capacity to improve non-heme iron absorption through a phenomenon called the “meat factor.” This effect shows that heme iron, even in trace amounts, can greatly increase the rates at which non-heme iron is absorbed; indeed, some studies have demonstrated that total absorption rose by 40% when heme and non-heme iron supplements were combined [38]. Starting from these results, the further focus of our study was to determine a concrete biological explanation to this enhanced uptake through the analysis of distinct iron uptake mechanisms at the cellular level.

At the levels of transport, storage, and excretion, iron metabolism is tightly controlled. On the intestinal enterocyte’s apical membrane, DMT1 absorbs non-heme iron from the diet [39], while heme iron is transported into the cells by HCP-1, a proton-coupled folate transporter [40]. Iron can be stored in cytosolic ferritin, which serves as a cellular iron storage, or used in metabolic activities. Regarding iron transport from the intestine to the bloodstream, the basal membrane’s ferroportin exports iron into the bloodstream [39]. The data on iron internalisation into intestinal cells revealed that all agents increased DMT1 levels, indicating a correct mechanism of iron uptake. Even in this case, the best results were obtained after stimulation with nutraceutical product 1, nutraceutical product 2, and the double administration of nutraceutical product 2, which induced an increase in DMT1 levels superior to that of all the other agents tested. However, these formulations also contain heme iron; thus, internalisation can also occur through HCP-1. Indeed, the data obtained revealed that only these three formulations could enhance HCP-1 levels, demonstrating that the heme iron present in nutraceutical product 1, nutraceutical product 2, and the double administration of nutraceutical product 2 was correctly internalised.

Ferritin concentrations are consistently low in patients with iron deficiency; indeed, low serum ferritin concentrations have been the diagnostic standard for iron deficiency [41]. It is now established that heme iron is absorbed more efficiently than non-heme iron, and this could result in a greater intracellular iron load in enterocytes. Further, following heme degradation, the increased availability of labile iron may enhance ferritin synthesis and storage. Indeed, while all the agents tested were able to increase ferritin levels compared to the control, indicating iron storage, nutraceutical product 1 induced the greatest increase in ferritin levels, indicating that a combination of heme and non-heme iron containing 18 mg of elemental iron could be the best formula among the tested products to increase ferritin levels. Finally, the amount of iron entering the body is then strictly controlled by the iron transporter ferroportin, which exports iron into the extracellular fluid [42]. The data obtained from ferroportin analysis indicated normal iron metabolism with all agents tested. Again, nutraceutical product 1, nutraceutical product 2, and the double administration of nutraceutical product 2 increased levels to a greater extent than all the other agents. Among them, nutraceutical product 1 exerted the greatest effect, although it was not statistically significant compared to double administration of nutraceutical product 2.

Therefore, according to the collected data, nutraceutical product 1 and nutraceutical product 2, as well as the double administration of nutraceutical product 2, considerably enhance the expression of tight junction proteins, thereby preserving the intestinal epithelial barrier’s functionality and even enhancing its integrity, as shown by the increase in TEER values. Furthermore, these findings suggest that the combination of non-heme and heme iron is beneficial for intestinal cells, helping to maintain and promote iron absorption and metabolism.

## 5. Conclusions

In conclusion, this in vitro investigation demonstrated that formulations containing both heme and non-heme iron can actively enhance intestinal iron absorption. This suggests the great potential of these combinations in novel dietary supplement regimens. The integrity of the intestinal barrier was preserved and strengthened by these combinations; this could be translated into enhanced product tolerability in the clinical setting. Additionally, the mechanism of iron absorption was examined using important indicators of iron metabolism, including DMT-1, HCP-1, ferritin, and ferroportin. In comparison to all other compounds examined, nutraceutical product 1, nutraceutical product 2, and the double administration of nutraceutical product 2 demonstrated superior overall performance. Thus, according to these in vitro data, the combination of heme and non-heme iron is a promising option for iron supplementation as an enhancer of iron absorption. However, to validate the in vitro data reported, in vivo investigations are necessary.

## Data Availability

The data presented in this study are available on request from the corresponding author (The Laboratory of Physiology carefully stores raw data to ensure permanent retention under a secure system).

## Abbreviations

The following abbreviations are used in this manuscript:

ANOVA	One-way analysis of variance
ATCC	American type culture collection
DMEM	Dulbecco’s modified eagle’s medium
DMEM-Adv	DMEM advance
DMT1	Divalent metal transporter 1
EMA	European Medicines Agency
FBS	Foetal bovine serum
FDA	Food and Drug Administration
HCP-1	Heme carrier protein-1
TEER	Trans-epithelial electrical resistance
TJ	Tight junction
WHO	World Health Organization
ZO-1	Zonula Occludens-1
